# Sumoylation of Kif18A plays a role in regulating mitotic progression

**DOI:** 10.1186/s12885-015-1226-9

**Published:** 2015-03-28

**Authors:** Feikun Yang, Yan Chen, Wei Dai

**Affiliations:** 1 Department of Environmental Medicine, New York University Langone Medical Center, 57 Old Forge Road, Tuxedo Park, NY 10987 USA; 2 Center for Drug Discovery, Northeastern University, 360 Huntington Avenue, Boston, MA 02115 USA; 3 Department of Biochemistry and Molecular Pharmacology, New York University Langone Medical Center, 57 Old Forge Road, Tuxedo Park, NY 10987 USA

**Keywords:** Kif18A, Sumoylation, Cell cycle, Mitosis, Motor protein, Microtubules

## Abstract

**Background:**

Kif18A, the kinesin-8 motor protein, plays an essential role in regulating alignment of bi-oriented chromosomes at the midzone during mitosis. Kinesin proteins, including Kif18A, are often deregulated in many types of cancers and are thought to play a critical role in cancer progression. However, little is known about the post-translational modifications of Kif18A and their effects on its biological activity.

**Methods:**

Kif18A was identified to be a SUMO2 acceptor by using Ni-IDA resin to precipitate proteins from cells stably expressing His_6_-SUMO2. To identify the potential lysine residues, multi-site directed mutagenesis together with transient transfection and Ni-IDA pull-down assay were carried out. The confocal time-lapse imaging and immunofluorescent staining were used to study the roles of SUMO2 modification on Kif18A’s activity during the cell cycle.

**Results:**

Kif18A is covalently modified by SUMO2 during the cell cycle, and its sumoylation peaks at metaphase and then rapidly decreases upon anaphase onset. Mutational analysis identifies multiple lysine residues (K148, K442, K533, K660 and K683) as potential SUMO acceptors. The functional studies reveal that sumoylation of Kif18A has little effect on protein stability and subcellular localization. However, compared with the wild-type control, ectopic expression of SUMO-resistant mutants of Kif18A results in a significant delay of mitotic exit. Confocal microscopy shows that cells expressing SUMO-resistant Kif18A display a compromised dissociation of BubR1 from kinetochores after anaphase onset.

**Conclusions:**

Our studies reveal that sumoylation functions as an unidentified form of post-translational modification that regulates Kif18A activity during mitotic progression.

**Electronic supplementary material:**

The online version of this article (doi:10.1186/s12885-015-1226-9) contains supplementary material, which is available to authorized users.

## Background

Proper equatorial alignment of all condensed chromosomes is an essential cellular process for preserving chromosomal stability during nuclear division. To this end, eukaryotic cells have evolved a system in which a set of conserved proteins monitor completion of chromosomal congression and regulate the dynamics of spindle microtubules at both spindle poles and kinetochores [1-3]. Increasing evidence indicates that KIF18A, the kinesin-8 molecular motor, plays an important role in regulating spindle microtubule dynamics and chromosome positioning during mitosis. As a plus-end directed motor, Kif18A inhibits polymerization dynamics of microtubules, thus suppressing kinetochore movements [4] and chromosome oscillations [5]. Depletion of Kif18A results in chromosome congression defects, which is at least partially mediated through destabilizing another plus-end directed motor protein CENP-E [6]. Mouse genetic study reveals that ablation of *KIF18A* causes complete sterility [7].

Kinesin proteins are often deregulated in many types of cancers and are thought to play a critical role in cancer progression [8-10]. For example, Kif18A is overexpressed in human breast cancer at both mRNA and protein levels, and the degree of Kif18A expression is associated with tumor grades, metastasis and survival [11]. Kif18A expression is up-regulated in colorectal tumors [12,13]. Ablation of Kif18A reduces cancer cell proliferation, migration and invasion [12], and promotes cell apoptosis through negative regulation of the PI3K-AKT signaling axis [13]. It has been also reported that Kif18A can be potentially served as a biomarker for diagnosing early stages of choloangiocarcinoma [14] and for identifying asbestosis patients at risk of developing lung cancer [15].

Post-translational modifications play important roles in regulating the activity of kinesin proteins. For example, kinesin light chain 1 of kinesin-1 is phosporylated at serine 460 by ERK and this phosporylation regulates its ability in cargo-binding and trafficking [16]. Kif2A, a microtubule depolymerase, is phosphorylated by Aurora B on multiple sites and the phosphorylation is important for the kinesin to function properly in cytokinesis [17,18]. Moreover, CENP-E, a member of kinesin-7 family, is modified by SUMO-2/3 and the modification is essential for its kinetochore localization during mitosis [19]. Furthermore, Kif18A is modified by phosphorylation and ubiquitination during mitosis and these modifications appear to play an important role in regulating degradation of Kif18A at anaphase [20-22].

Given that sumoylation plays an essential role in regulating mitotic proteins [23], we asked whether Kif18A was modified by sumoylation and whether the modification affected its activity in mitosis. We found that Kif18A was preferentially modified by SUMO2 and that the modification was closely associated with mitotic progression. Site-directed mutagenesis coupled with ectopic expression revealed that several lysine residues (K148, K442, K533, K660 and K683) were potential SUMO2 acceptors. Expression of a SUMO-deficient Kif18A mutant, but not the wild-type counterpart resulted in a significant delay in mitotic exit. Therefore, our combined study reveals a new type of post-translational mechanism that regulates Kif18A’s function in mitosis.

## Methods

### Cell culture

HeLa and HEK293T cells were cultured in DMEM supplemented with 10% fetal bovine serum (FBS, Invitrogen) and antibiotics (100 μg/ml of penicillin and 50 μg/ml of streptomycin sulfate, Invitrogen) at 37°C under 5% CO_2_.

### Cell cycle synchronization

HeLa cells were synchronized at the G_1_/S boundary by double-thymidine blocks. Briefly, cells were treated with 2 mM thymidine for 18 h followed by a 9 h release; the cells were treated with 2 mM thymidine for another 18 h and then released into the cell cycle for various times. Mitotic shake-off cells were obtained from gentle tapping of cell culture plates treated with nocodazole (40 ng/ml) or taxol (40 nM) (Sigma-Aldrich) for 16 h. In some experiments, mitotic cells were rinsed and cultured in fresh medium for indicated times before harvesting for various analyses.

### Antibodies

Kif18A antibodies were purchase from Bethyl Laboratories LLC. Antibodies to HA, Flag and β-actin were purchased from Cell Signaling Technology Inc. Rabbit polyclonal antibodies to BubR1 were developed in the laboratory. GFP antibodies were purchased from Santa Cruz Biotechnology. Mouse anti-SUMO2/3 antibodies were kindly provided by Dr. Michael J. Matunis (Johns Hopkins University).

### Plasmids, mutagenesis, and transfection

Full-length wild-type human *KIF18A* cDNA with HA-his tag was subcloned into pcDNA3 plasmid or a GFP-expression plasmid. Potential SUMO targeting lysine mutants were generated using the QuickChange Lightning Multi Site-directed Mutagenesis kit (Stratagene). Individual mutations were confirmed by DNA sequencing. SENP-1 and its mutant expression plasmids were kindly provided by J. Cheng [24]. Plasmid transfection was carried out using Fugene HD according to instructions provided by the supplier (Roche).

### RNA interference

Small interfering RNAs (siRNAs) of human KIF18A were synthesized from Dharmacon which corresponded to the following sequences: 5′ACA GATTCGTGATCTCTTA3′, which is known to silence human *KIF18*A [6]. Briefly, cells seeded at 60% confluency in an antibiotic-free culture medium were transfected using Lipojet^™^ (Signagene) with siRNA duplexes at a final concentration of 200 pM for 48 hours. Firefly (Photinus pyralis) luciferase siRNAs (5′UUCCTACGCTGAGTACTTCGA3′, GL-3 from Dharmacon) were served as negative control.

### Western blot

SDS-PAGE was carried out using the mini gel system from Bio-Rad. Proteins were transferred to PVDF membranes. After blocking with TBST containing 5% nonfat dry milk for 1 h, the membranes were incubated with primary antibodies overnight at 4°C followed by incubation with horseradish peroxidase-conjugated secondary antibodies for 1 h at room temperature. After thorough washing the membranes with TBST buffer, signals were developed with an enhanced chemiluminescent system (Pierce).

### Pull-down analysis

HeLa cells transfected with indicated plasmids or stably expressing His_6_ -tagged SUMO-2 were lysed in a lysis buffer [50 mM Na_2_HPO_4_/NaH_2_PO_4_ (pH 7.4), 300 mM NaCl, 8 M urea, 0.2% Triton X-100] supplemented with 20 mM imidazole. Ni^2+^-IDA-agarose resin (Clontech) was then added to the cell lysates and incubated at room temperature for 3 h. The resin was washed 3 times at room temperature with the lysis buffer supplemented with 40 mM imidazole. After washing, His_6_ -tagged proteins were eluted in the lysis buffer containing 300 mM imidazole. Samples were then blotted with individual antibodies.

### Fluorescence microscopy

Fluorescence microscopy was essentially performed as described [23]. Briefly, HeLa cells seeded on chamber slides were transfected with indicated expression constructs for 48 h. At the end of transfection, cells were fixed with 4% paraformaldehyde in PBS for 20 min at room temperature. After permeabilization using 0.5% Triton X-100 in PBS for 20 min, cells were incubated with 2% bovine serum albumin (BSA) in PBS for 1 h followed by incubation overnight with the antibody to BubR1. Cells were stained with Alex Fluor 555-conjugated goat anti-rabbit IgGs (Invitrogen) for 1 h. Cellular DNA was finally stained with 4′,6-diamidino-2-phenylindole (DAPI, Molecular Probe, Eugene, OR). Fluorescence signals were detected on a Leica TCS SP5 confocal microscope.

### Statistical analysis

Student’s *t* test was used to evaluate significance of differences between two groups. A P value <0.05 was considered statistically significant.

## Results

**Figure 1:**
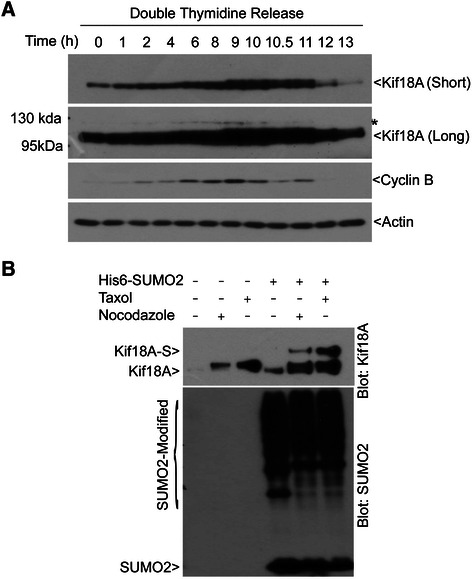
**SUMO2 modification of Kif18A at mitosis. (A)** HeLa cells were subjected to double thymidine treatment as described in Materials & Methods. Cell pellets were lysed in 8 M urea. Equal amount of cell lysates were blotted for antibodies against Kif18A, cyclin B1, and β-actin. Kif18A blots of both short and long exposure are shown. Asterisk (*) indicates the Kif18A related signals. **(B)** Parental HeLa cells and HeLa cells stably expressing transfected His_6_ -SUMO2 were treated with nocodazole (40 ng/mL) or taxol (40 nM) for 16 h and then lysed in 8 M urea. Equal amounts of cell lysates were used for Ni-IDA pull-down analysis. The precipitates were blotted for Kif18A and SUMO2. Kif18A-S denotes potentially SUMO-modified Kif18A.

To study post-translational modifications of Kif18A and their potential function in regulating mitotic progression, synchronized HeLa cells through the double-thymidine block were released into the cell cycle for various times. Immunoblotting analysis revealed that Kif18A levels gradually increased during the release, peaking around 10 h before returning to the basal level (Figure 1A). Intriguingly, as cells entered mitosis as indicated by cyclin B1 levels, a slower mobility band immunoreactive to the Kif18A antibody was present (Figure 1A, Asterisk). The slow mobility signal also peaked around 10 h post the double thymidine release and became barely detectable 1 h after exiting from mitosis. Oscillation of Kif18A-specific signals suggests a role of the kinesin and its modified form in regulating mitotic progression.

The molecular mass of the slow mobility band of Kif18A was about 125 kDa, which is 15 kDa larger than the non-modified form. Given the major size difference between the basal and modified forms, we speculated that it might be caused by SUMO modification. To test this hypothesis, we took advantage of the cell lines stably expressing His_6_ -SUMO2 [25]. Cells were arrested at mitosis by nocodazole or taxol for 16 h before harvesting. Equal amounts of cell lysates were used for Ni-IDA resin pull-down analysis and the precipitates were blotted for antibodies against Kif18A and SUMO2. A slower mobility band immunoreactive to Kif18A antibody was detected in SUMO2-expressing cells in mitotic cells but not in asynchronized cells. This band was not present in parental cells arrest at mitosis. These observations suggest that Kif18A is targeted by SUMO2 at mitosis. Interestingly, taxol enhanced the Kif18A signal to a greater extent than that of nocodazole, which is likely due to microtubule stabilization by taxol that triggers a significant plus-end accumulation of Kif18A [26]. Unmodified Kif18A was also detected in the pull-down precipitates, which could be derived from its electrostatic interaction with the Ni-IDA resin or the proteins binding to the resin.

**Figure 2:**
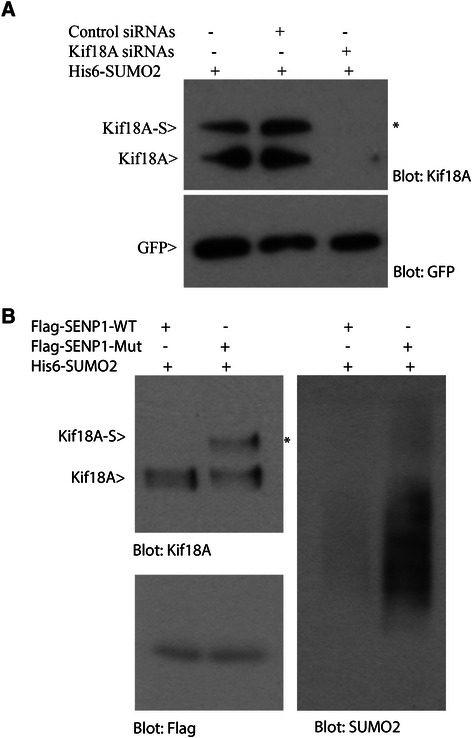
**Kif18A is SUMO2-modified. (A)** HeLa cells stably expressing His_6_ -SUMO2 were co-transfected with Kif18A (or control) siRNAs and a plasmid construct expressing His_6_ -GFP for 48 h. Transfected cells were then treated with 40 nM taxol for 16 h. At the end of treatment, cells were lysed in 8 M urea. Equal amounts of cell lysates were subjected to Ni-IDA pull-down analysis. Pull-down proteins were then blotted for Kif18A and GFP. Kif18A-S denotes SUMO2-modified Kif18A. **(B)** HeLa cells stably expressing His_6_ -SUMO2 were transfected with a plasmid expressing FLAG-tagged SENP-1 or enzymatically defective SENP1 (SENP-1-Mut) for 48 h. Transfected cells were then treated with 40 nM taxol for 16 h. At the end of treatment, cell pellets were lysed in 8 M urea. Equal amounts of cell lysates were subjected to Ni-IDA pull-down analysis. Pull-down proteins were then blotted for Kif18A, Flag, and SUMO2. Notably, Flag-SENP1 was precipitated by Ni-IDA resin due to its histidine-rich property.

To further confirm that the slower mobility band is Kif18A-specific, we transfected His_6_ -SUMO2 cells with siRNAs to Kif18A or luciferease. His_6_ -GFP was used for co-transfection to monitor transfection and pull-down efficiency. Transfected cells were treated with or without taxol for 16 h and equal amounts of cell lysates were subjected to pull-down analysis. As shown in Figure 2A, Kif18A siRNAs, but not control siRNAs, almost completely depleted the slow mobility band, indicating that the signal was Kif18-specific.

SUMO modification is a reversible process, and de-conjugation of SUMO from targeted proteins is catalyzed by sentrin-specific isopeptidases. In vertebrates, six SUMO-specific isopeptidases, including SENP1, SENP2, SENP3, SENP5, SENP6, and SENP7, have been reported [27]. To further confirm Kif18A is modified by SUMO2, His_6_ -SUMO2-expressing cells were transiently transfected with a plasmid construct expressing either FLAG-tagged wild-type SENP1 or its enzymatically inactive counterpart (SENP1-Mut). The transfected cells were then treated with taxol for 16 h. Ni-IDA pull-down precipitates were blotted for Kif18A, FLAG and SUMO2. As shown in Figure 2B, expression of FLAG-SENP1 largely eliminated the slow mobility band that was immunoactive to Kif18A antibody. However, the mutant SENP1 was not effective in suppressing the signal. SENP1 could also be precipitated by Ni-IDA resin due to its histidine-rich property. Thus, expression of both SENP1 and its mutant was confirmed by blotting with the anti-FLAG antibody (Figure 2B). Combined, these results strongly support the notion that Kif18A is modified by SUMO2 at mitosis.

**Figure 3:**
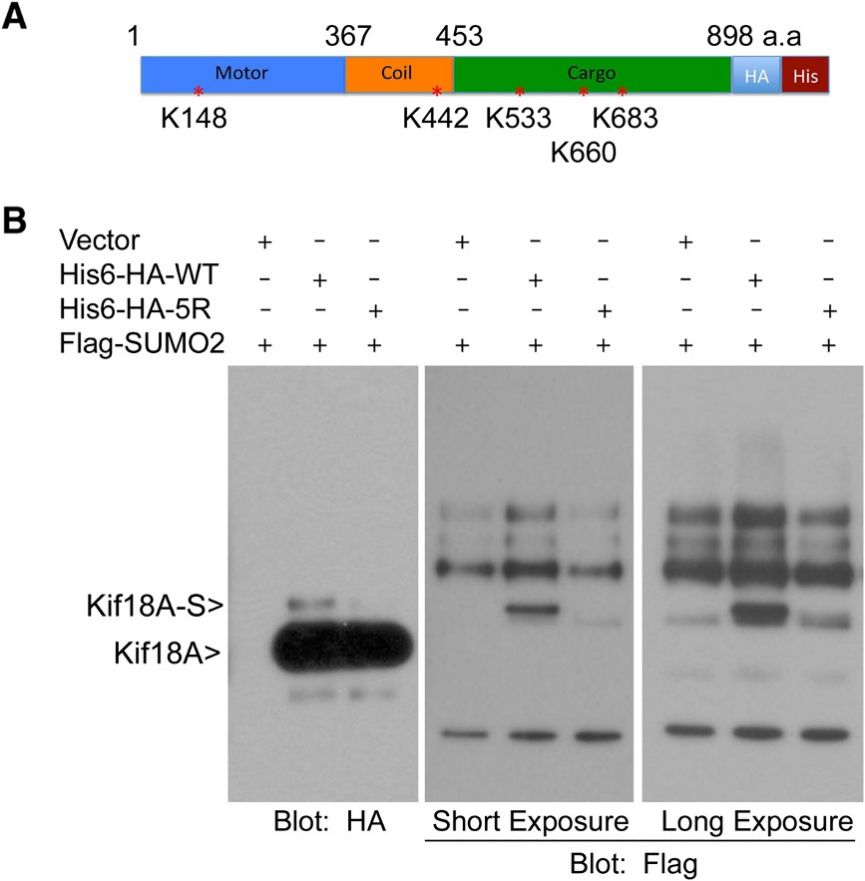
**Identification of Kif18A sumoylation sites. (A)** Schematic representation of lysine residues of wild-type human Kif18A. Key lysine residues subjected to mutational analsyis are indicated. HA and His_6_ tags are fused in-frame at the C-terminus. Three major functional domains of Kif18A are also shown. **(B)** HeLa cells were transfected with plasmids expressing His_6_ -HA-tagged Kif18A (His_6_ -HA-WT) or the mutant protein with 5 lysine residues (K148, K442, K533, K660 and K683) replaced with arginines (His_6_ -HA-5R) for 48 h. Flag-SUMO2 was also used for co-transfection. Transfected cells were then treated with 40 nM taxol for 16 h. At the end of treatment, cell pellets were lysed in 8 M urea. Equal amounts of cell lysates were subjected to Ni-IDA pull-down analysis. Pull-down proteins were blotted for HA and Flag signals. Flag blots of both short and long exposure are shown.

To identify the potential lysine residue (s) for sumo modification, we analyzed Kif18A amino acid sequences for optimal sumoylation using the criteria available at Abgent Inc. Five lysines sites (K148, K442, K533, K660 and K683) with the highest scores were subjected to mutagenic analysis. The relative position of these sites to other domains is shown in Figure 3A. HeLa cells were co-transfected for 48 h with a SUMO2- construct and a construct expressing His_6_ -HA-tagged wild-type Kif18A (His_6_-HA-WT) or Kif18A with 5 lysine residues mutated into arginines (His_6_-HA-5R). After treatment with Taxol for 16 h, cells were lysed and equal amounts of cell lysates were subjected to pull-down analysis using Ni-NDA resin. Immunoblotting with antibody against the HA tag showed major bands for both ectopically expressed His_6_-HA-WT and its mutant counterpart (Figure 3B). Of great importance is that an extra slower mobility band that was immunoreactive to HA antibody was detected in cells expressing His_6_-HA-WT, but not His_6_-HA-5R. Furthermore, immunoblotting with the antibody to FLAG revealed a specific band migrated at the same position as the one detected by the HA tag antibody. These results not only confirmed that Kif18A was modified by SUMO2 at mitosis but also indicated that K148, K442, K533, K660 and/or K683 were potential acceptors for SUMO2. The molecular difference between SUMO2-modified and unmodified Kif18A was about 15 kDa, which suggests mono sumoylation. On the other hand, we were unable to identify the single lysine residue for the modification as mutation of any of these lysine residues alone failed to abolish the signal (see Additional file 1).

**Figure 4:**
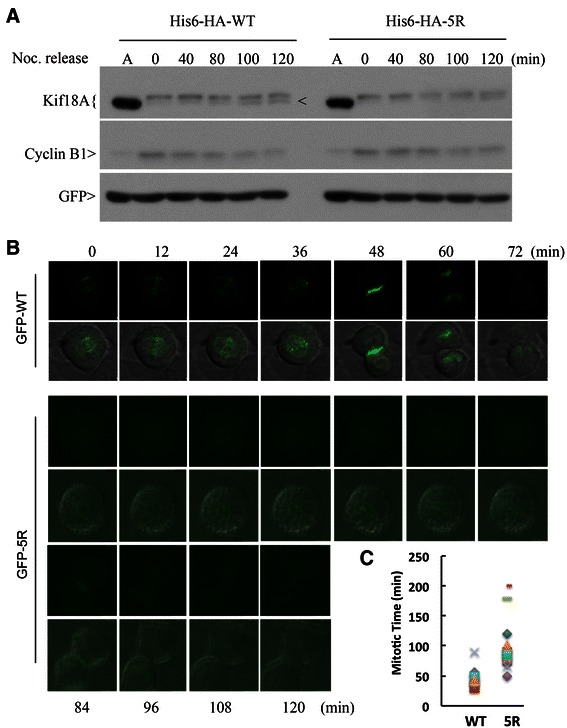
**Sumoylation-resistant Kif18A mutant induces a mitotic delay. (A)** HeLa cells were co-transfected with a plasmid expressing His_6_ -HA-WT or His_6_ -HA-5R and a plasmid construct expressing His_6_ -GFP for 36 h. Transfected cells were then treated with nocodazole (40 ng/mL) for 14 h, after which mitotic cells were collected by shake-off. Mitotic cells were then released into fresh medium. Cells were collected at various times of release and lysed in 8 M urea. Equal amounts of cell lysates were blotted for HA, cyclin B1, and GFP. Asterisk indicates phosphorylated form of His_6_ -HA-Kif18A. Lane A denotes lysates from asynchronized cells to show the unmodified form of His_6_ -HA-Kif18A. **(B)** HeLa cells transfected with GFP-WT or GFP-5R were subjected to time-lapse confocal microscopy analysis. The video-graphic process started when significant plus-end accumulation of GFP signals was observed. **(C)** Quantitative analysis of mitotic time of cells as shown in B. Data were summarized from three independent experiments (WT: n = 13; 5R: n = 13).

Sumoylation plays an important role in regulating stability and subcellular localization of targeted proteins [28,29]. However, this does not seem to be the case for Kif18A as there was no significant difference in the half-life between His_6_-HA-WT and His_6_-HA-5R (see Additional file 2). Since Kif18A sumoylation peaked at metaphase and rapidly decreased thereafter (Figure 1A), we then asked whether Kif18A sumoylation might be involved in regulating mitotic exit. Kif18A exists in unmodified form in interphase, and undergoes dynamic phosphorylation/de-phosphorylation during mitosis (Figure 1 and see Additional file 3). Dephosphorylation of His_6_ -HA-WT took place within 40 minutes upon nocodazole release (Figure 4A, arrow). However, compared with His_6_-HA-WT, sumoylation-resistant mutant His_6_-HA-5R appeared to be dephosphorylated at a slower pace, which was accompanied by slower decline of cyclin B1 (Figure 4A and see Additional file 4). These observations suggest that cells expressing His_6_-HA-5R exhibit delayed mitotic exit. To further confirm that sumoylation of Kif18A plays a role in regulating mitotic progression, HeLa cells ectopically expressing GFP-tagged Kif18A-WT (GFP-WT) or its mutant counterpart Kif18A-5R (GFP-5R) were examined via time-lapse confocal microscopy. We observed that both GFP-WT and GFP-5R exhibited normal microtubule plus-end localization in metaphase cells (Figure 4B), which is consistent with a previous report that Kif18A strongly accumulates at microtubule plus-end during metaphase but not in prometaphase [1,4]. However, compared to GFP-WT, the majority of GFP-5R expressing cells exhibited a prolonged mitotic exit (98 ± 44 min vs. 47 ± 15 min as shown in Figure 4C).

**Figure 5:**
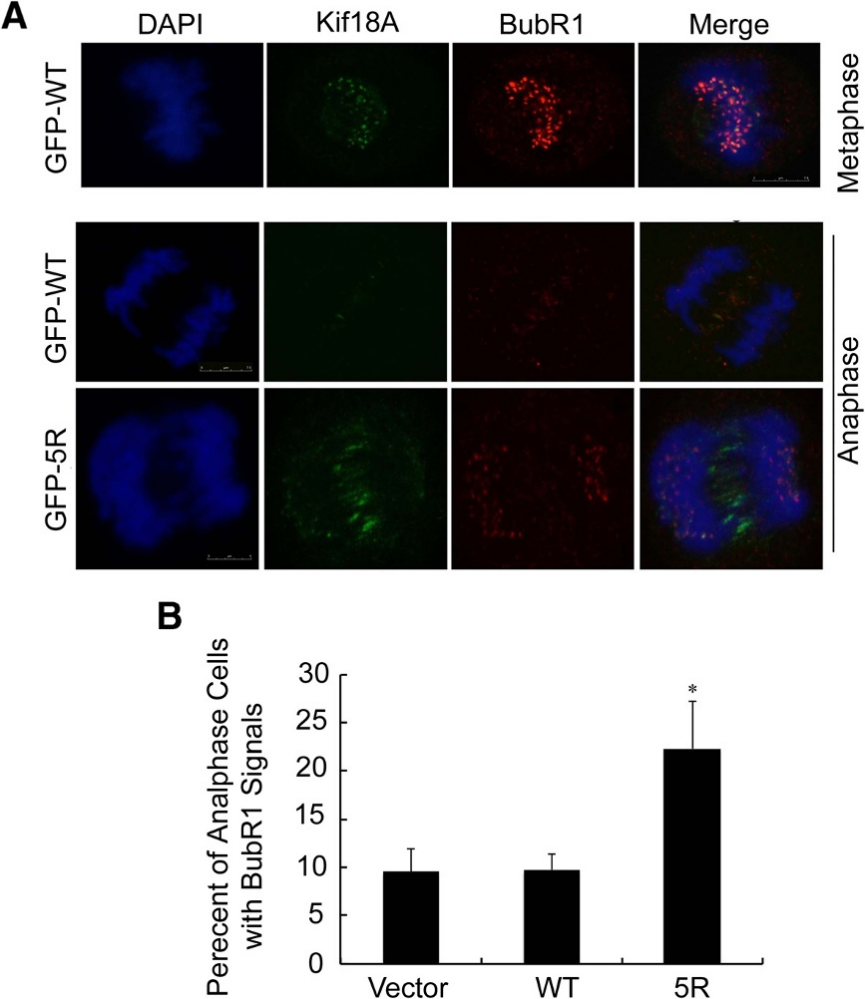
**Expression of sumoylation-resistant Kif18A induces aberrant BubR1 localization in anaphase cells. (A)** HeLa cells were transfected with a plasmid expressing GFP-WT for 48 h, after which cells were fixed and stained with the antibody against BubR1 (red). DNA was stained with DAPI (blue). Representative metaphase cell images are shown in the upper panel. In the lower panel, HeLa cells transiently expressing GFP-WT or GFP-5R were fixed and stained with the BubR1 antibody (red). DNA was stained with DAPI (blue). Representative anaphase cell images are shown. **(B)** Percentage of anaphase cells with aberrant (signals in anaphase) BubR1 localization were recorded and plotted. Data were summarized from three independent experiments (WT, n = 85; 5R, n = 94; vector, n = 76). Asterisk indicates statistically significant difference between mutant 5R and WT (or vector).

Our previous work implies that Kif18A may interact with BubR1, a spindle assembly checkpoint component, at the mitotic stage because both proteins are associated with CENP-E [6]. Indeed, GFP-Kif18A accumulated around the kinetochore region where BubR1 signals were also detected in metaphase cells ectopically expressing GFP-WT (Figure 5A, upper panel), suggesting a physical and functional interaction between these two molecules. To understand the underlying molecular mechanism responsible for the delayed mitotic exit in cells expressing GFP-5R, we determined the dissociation of BubR1 from kinetochore using fluorescence microscopy. HeLa cells transiently transfected with GFP-WT and its sumo-resistant counterpart for 36 h were fixed and stained with the antibody to BubR1. As expected, BubR1 was barely detectable at the kinetochores after the anaphase onset in control cells or cells expressing GFP-WT (Figure 5A & B). However, a significant fraction of GFP-5R-expressing cells displayed persistent kinetochore localization of BubR1 at anaphase and telophase stages, strongly suggesting that Kif18A sumoylation may regulate the removal of BubR1 from the kinetochores and compromise its inactivation. Of note, GFP-5R was also detected in the region where BubR1 signal persisted even after the apparent anaphase onset (Figure 5A, lower panel).

## Discussion

In this study we report that a fraction of Kif18A is covalently modified by SUMO2 when cells enter the mitotic stage. Kif18A sumoylation is a transient event as it is rapidly desumoylated upon the anaphase onset. Kif18A mutant with lysines 148, 442, 533, 660 and 683 replaced with arginines largely abolished its sumoylation during mitosis, strongly suggesting the involvement of these residues in mediating SUMO modification. Functional studies reveal that Kif18A sumoylation regulates mitotic progression as ectopic expression of sumoylation-resistant Kif18A mutant significantly delays mitotic exit. Moreover, sumoylation also plays a role in the removal of BubR1 from the kinetochores at the anaphase onset, thus participating in the checkpoint control.

Several studies have shown that at the onset of mitosis many important proteins are SUMO-modified, which is thought to function in the maintenance of mitotic chromosome structures [30-32]. It has also been reported that sumoylation is essential for the proper function of inner centromeric proteins, as well as components of outer kinetochore and fibrous corona [19,33,34]. However, the role of sumoylation in the regulation of kinesin motor proteins during the cell cycle remains largely unknown. Kif18A plays an important role in chromosome congression by suppressing chromosome movements [4]. Consistent with previous observations on both endogenous [1,4] and ectopically expressed venus-tagged Kif18A [21], GFP-Kif18A localizes along spindle microtubules in prometaphase cells (unpublished observation) and then exhibits as a comet-like gradient along kinetochore microtubules with the strongest signal detected at the plus-end. Moreover, after the anaphase onset Kif18A re-distributes to the midzone of the cell, as well as chromatin regions, suggesting that it may play a role in mid-body formation and cytokinesis. In agreement with previous study [4], expression of GFP-WT did not cause an obvious mitotic delay or disrupt chromosome alignment. When cells were transfected with SUMO-resistant GFP-5R, similar subcellular localization patterns were observed, indicating that sumoylation does not affect the plus-end localization of Kif18A. On the other hand, from both time-lapse microscopy and PFA fixed samples, we did not see apparent defects in chromosome alignment between cells expressing GFP-WT and GFP-5R, indicating that Kif18A sumoylation does not regulate the capture of microtubules to the kinetochores and the movement of chromosomes during congression. However, GFP-5R expressing cells displayed prolonged mitotic exit, suggesting that Kif18A sumoylation may play a role in regulating segregation of sister centromeres/chromosomes. Indeed, previous studies have shown that Kif18A directly regulates kinetochore fiber dynamics, thus controlling the attachment between kinetochores and microtubules [2,35,36]. Moreover, Kif18A physically interacts with kinetochore fibrous corona components CENP-E and BubR1 during mitosis [6], consistent with its role in regulating the dynamic connections between kinetochore and spindle microtubules.

It is known that BubR1 not only inhibits the activity of anaphase-promoting complex/cyclosome (APC/C) but also monitors kinetochore activities that depend on the kinetochore motor CENP-E [37]. Kif18A sumoylation can potentially affect the switch rate and velocity of kinetochore/chromosome oscillations at metaphase, thus affecting the tension across spindle poles and delaying mitotic progression. It has been shown that Kif18A attenuates centromere movements and increases the proportion of time that centromeres spend in a slow velocity state during both directional switches and persistent movements [4,38]. Expression of GFP-WT at metaphase suppresses kinetochore oscillatory movements through its motor activity. Moreover, the velocity of poleward anaphase movements is monitored by Kif18A [4]. It will be of interest to know whether sumoylation regulates the activity of Kif18A in controlling kinetochore microtubule dynamics.

Kif18A is up-regulated in several types of tumors and its expression is closely associated with the tumor grade, metastasis, and survival [11,13,14]. Consistent with its potential oncogenic role, depletion of Kif18A inhibits cancer cell growth both *in vitro* and *in vivo* [11]. Our current study shows that Kif18A expression is regulated in a cell cycle–dependent manner. Kif18A level is highest during mitosis and gradually declined after mitotic exit. Moreover, Kif18A sumoylation peaks at metaphase, after which its level is rapidly reduced. Thus, Kif18A sumoylation appears to be independent of the total protein level because its desumoylation takes place before the degradation of Kif18A (Figure 1A) [21,22]. Deregulation in the SUMO pathway is believed to contribute to the oncogenic transformation by affecting the balance of sumoylation/desumoylation on various oncoproteins and tumor suppressors [39-43]. The delayed mitotic exit of cells expressing SUMO-resistant GFP-5R suggests that SUMO proteins can be developed as a potential target for cancer therapy.

## Conclusions

Our study demonstrates that post-translational modification via SUMO2 regulates Kif18A activity during mitotic progression. As de-regulation of Kif18A plays critical roles in tumor progression, the SUMO regulatory network may be a potential target for cancer intervention.

## Additional files

Additional file 1: **Identification of potential lysine residues for Kif18A sumoylation.** HeLa cells stably expressing his6-SUMO2 were transiently transfected with plasmids expressing His_6_ -HA--tagged Kif18A (His_6_ -HA-WT) or the mutant protein with single lysine residue replaced with arginine (K148R, K442R, K533R, K660R and K683R) or the mutants with all 5 lysine residues replaced with arginines (5R) for 48 h. Transfected cells were then treated with 40 nM taxol for 16 h. At the end of treatment, cell pellets were lysed in 8 M urea. Equal amounts of cell lysates were subjected to Ni-IDA pull-down analysis. Pull-down proteins were blotted for HA and SUMO2 signals.
Additional file 2: **Sumoylation does not affect Kif18A protein stability.** Non-transfected HeLa cells or cells transfected with either His_6_-HA-WT or His_6_-HA-5R for 48 h were randomly but eaqually split into 5 dishes followed by treatment with 20ug/mL cycloheximide (CHX) for indicated times. Cells were harvested and lysed in 8 M urea. Equal amount of cell lysates were blotted for Kif18A, HA and actin signals as indicated.
Additional file 3: **Phosphorylation of Kif18A in mitosis.** HeLa cells (A) or HeLa cells transfected with His_6_-HA-WT plasmids (B) for 48 h were treated with 40nM taxol for 16 h, and then lysed on ice for 15 min in 1x NEB buffer for PMP supplemented with 1% triton X-100 and 1 mM MnCl_2_. After centrifugation, supernatant with 50ug of proteins was incubated with 400 units of lambda protein phosphatase (PPtase, New England Biolabs Inc) at 30C for 30 min. Equal amount of protein were blotted for Kif18A, HA and actin signals.
Additional file 4: **Delayed degradation of cyclin B1 in mitotic cells transfected with His**_**6**_**-HA-5R plasmids.** Quantitative analysis of relative cyclin B1 level as shown in Figure 4A was graphed. Data were summarized from 3 replicates.
