# Supplementary figures and images for: Sumoylation of Kif18A plays a role in regulating mitotic progression
Source: BMC Cancer. 2015 Mar 28;15:197. doi: 10.1186/s12885-015-1226-9 (PMC4389313; doi:10.1186/s12885-015-1226-9)

Additional file 1

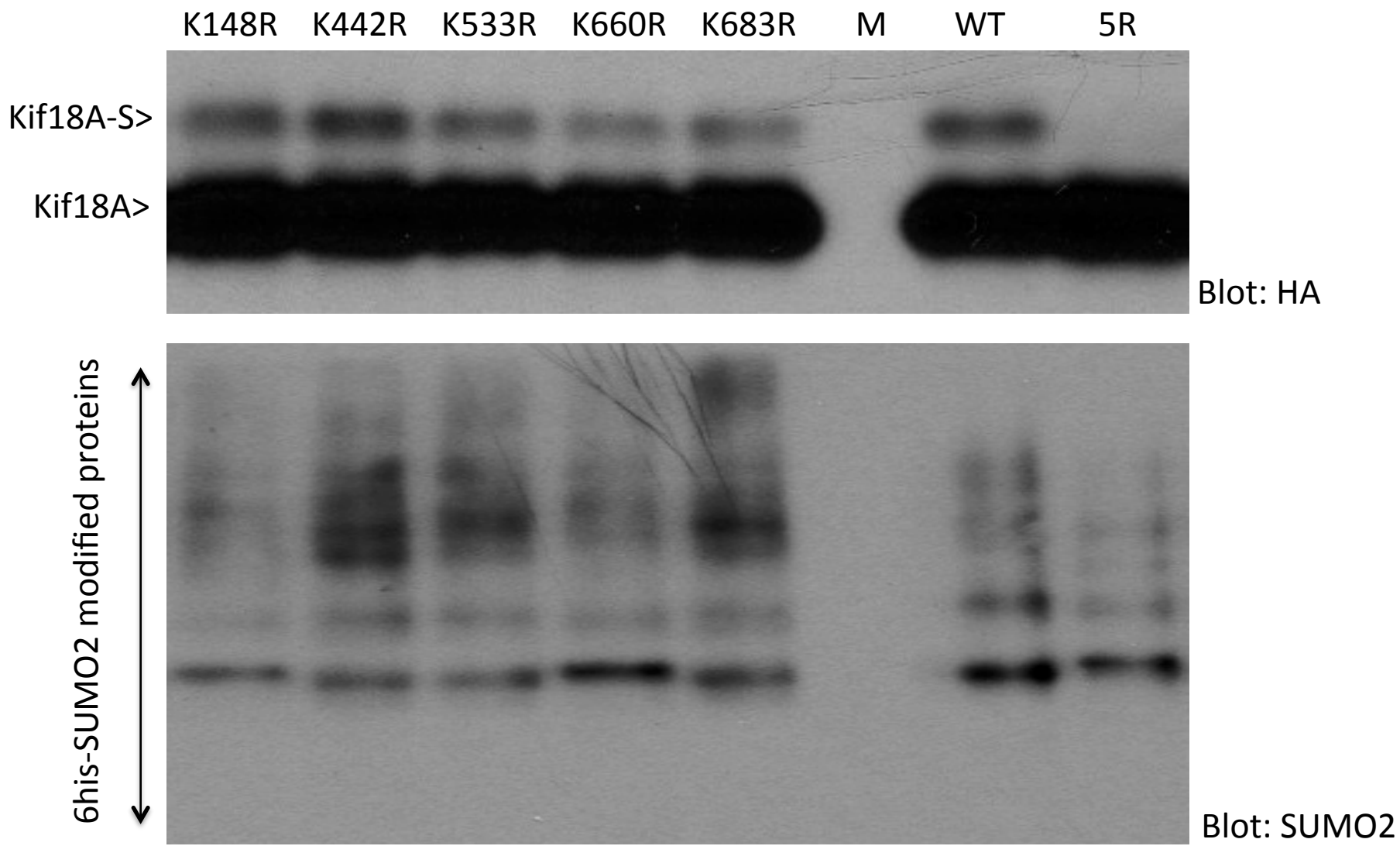

Supplement: Additional file 1: — Identification of potential lysine residues for Kif18A sumoylation. HeLa cells stably expressing his6-SUMO2 were transiently transfected with plasmids expressing His6 -HA--tagged Kif18A (His6 -HA-WT) or the mutant protein with single lysine residue replaced with arginine (K148R, K442R, K533R, K660R and K683R) or the mutants with all 5 lysine residues replaced with arginines (5R) for 48 h. Transfected cells were then treated with 40 nM taxol for 16 h. At the end of treatment, cell pellets were lysed in 8 M urea. Equal amounts of cell lysates were subjected to Ni-IDA pull-down analysis. Pull-down proteins were blotted for HA and SUMO2 signals. [file 12885_2015_1226_MOESM1_ESM.pdf]

## Additional file 2

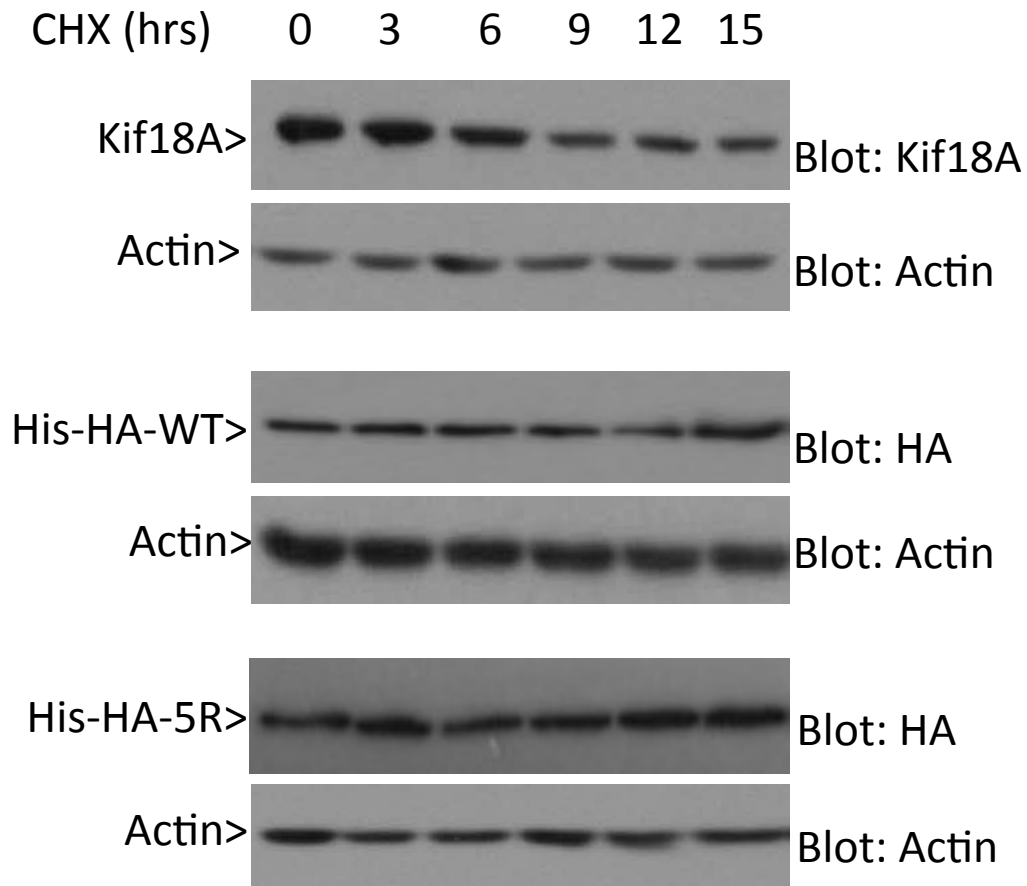

Supplement: Additional file 2: — Sumoylation does not affect Kif18A protein stability. Non-transfected HeLa cells or cells transfected with either His6-HA-WT or His6-HA-5R for 48 h were randomly but eaqually split into 5 dishes followed by treatment with 20ug/mL cycloheximide (CHX) for indicated times. Cells were harvested and lysed in 8 M urea. Equal amount of cell lysates were blotted for Kif18A, HA and actin signals as indicated. [file 12885_2015_1226_MOESM2_ESM.pdf]

## Additional file 3

A

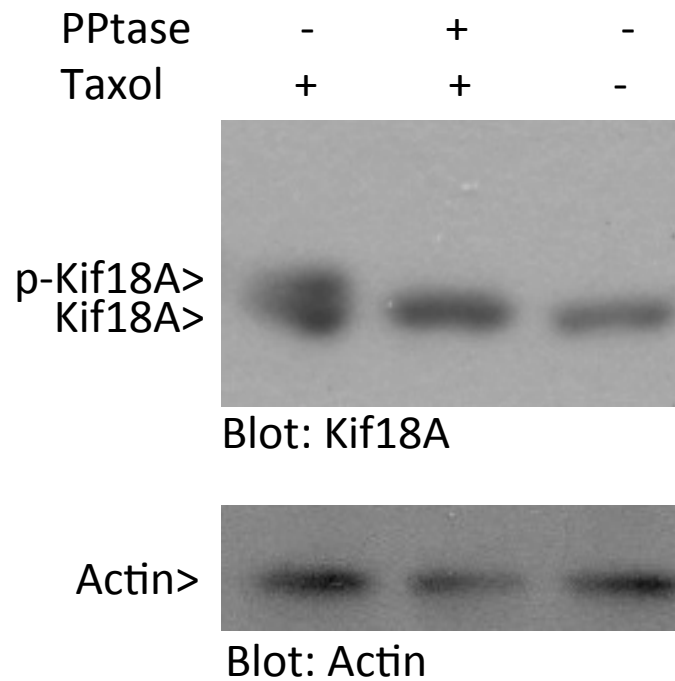

B

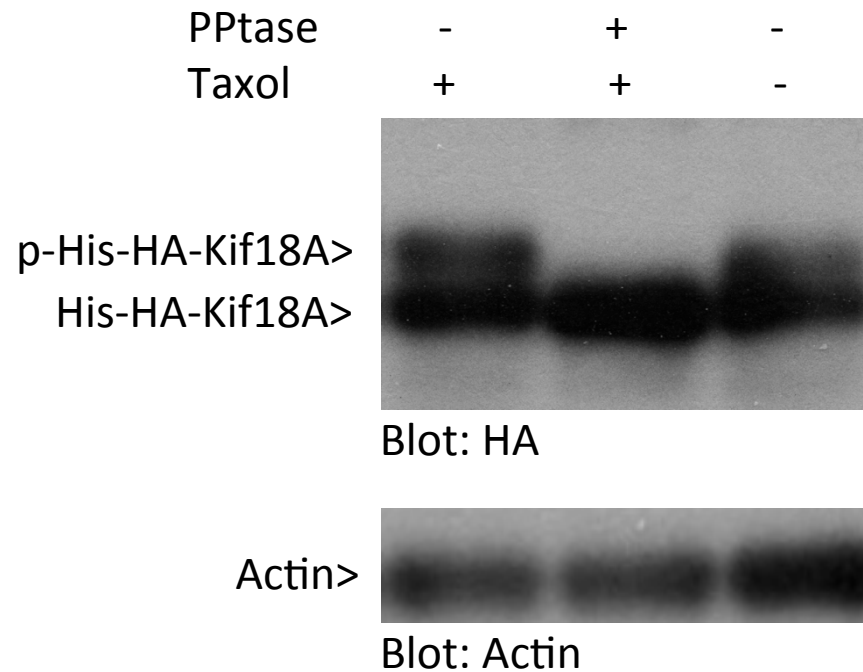

Supplement: Additional file 3: — Phosphorylation of Kif18A in mitosis. HeLa cells (A) or HeLa cells transfected with His6-HA-WT plasmids (B) for 48 h were treated with 40nM taxol for 16 h, and then lysed on ice for 15 min in 1x NEB buffer for PMP supplemented with 1% triton X-100 and 1 mM MnCl2. After centrifugation, supernatant with 50ug of proteins was incubated with 400 units of lambda protein phosphatase (PPtase, New England Biolabs Inc) at 30C for 30 min. Equal amount of protein were blotted for Kif18A, HA and actin signals. [file 12885_2015_1226_MOESM3_ESM.pdf]

## Additional file 4

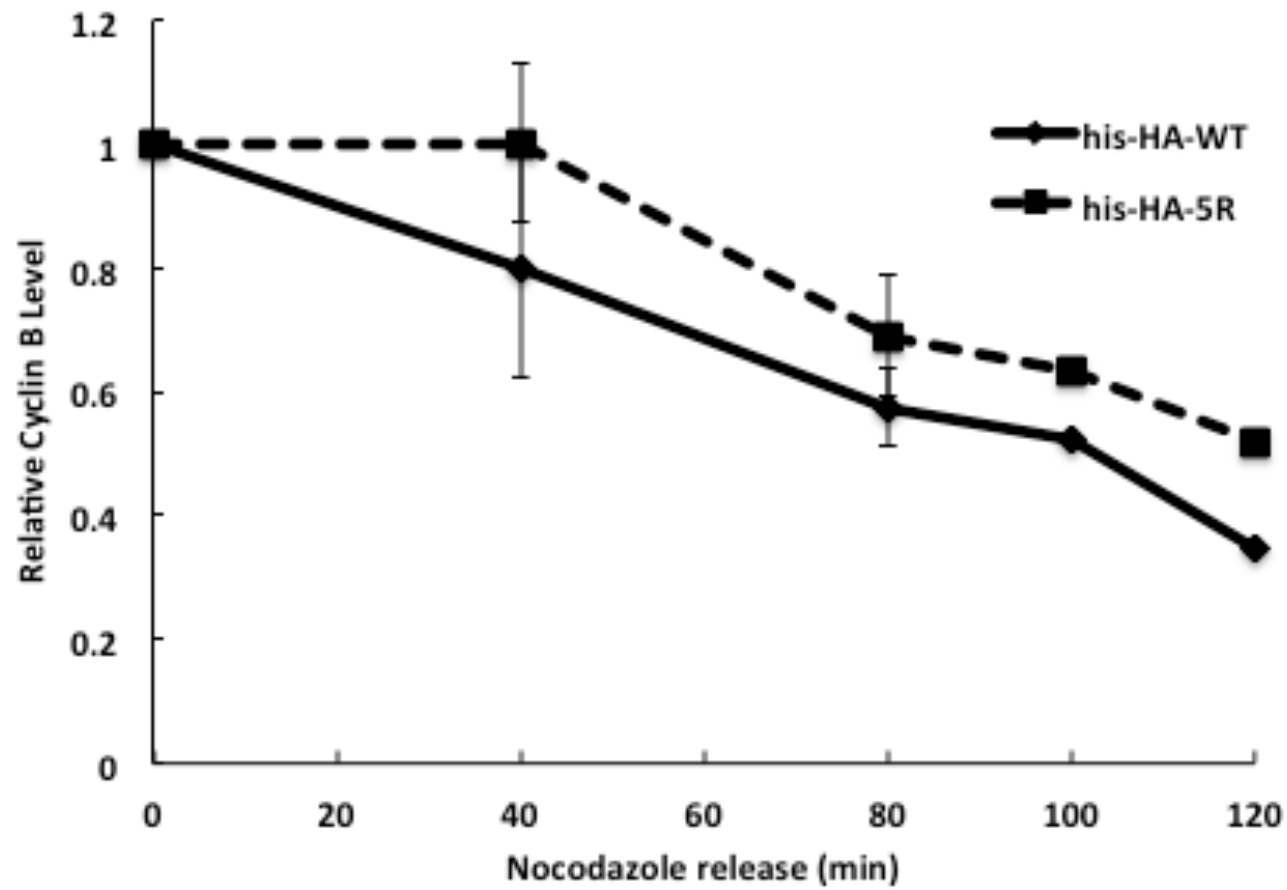

Supplement: Additional file 4: — Delayed degradation of cyclin B1 in mitotic cells transfected with His6-HA-5R plasmids. Quantitative analysis of relative cyclin B1 level as shown in Figure 4A was graphed. Data were summarized from 3 replicates. [file 12885_2015_1226_MOESM4_ESM.pdf]
